# Sertoli cells-only syndrome: current clinical approaches and ongoing research trends

**DOI:** 10.3389/fendo.2025.1715642

**Published:** 2025-12-19

**Authors:** Elena Eugeni, Iva Arato, Francesca Mancuso, Stefano Brancorsini, Giovanni Luca, Sieglinde Kofler

**Affiliations:** 1 Department of Internal Medicine, Meran Hospital Südtiroler Sanitätsbetrieb (SABES), Merano, Italy; 2 Department of Medicine and Surgery, University of Perugia, Perugia, Italy; 3 International Biotechnological Center for Endocrine, Metabolic and Embryo-Reproductive Translational Research (CIRTEMER), Department of Medicine and Surgery, University of Perugia, Perugia, Italy; 4 Department of Medicine and Medical Specialties, Division of Endocrinology, Metabolic Diseases, Terni, Italy

**Keywords:** Sertoli cells-only syndrome, SCOS, NOA, ART, spermatogenesis, Sertoli cells

## Abstract

Sertoli Cells-Only Syndrome (SCOS), also known as Del Castillo syndrome or germ cell aplasia, is the most frequent cause of non-obstructive azoospermia, being found in 26-57% of patients affected by this condition. Although up to 10% of infertile males seeking medical attention are affected by SCOS and almost 80 years have already passed since this challenging syndrome was first described, therapeutic approaches to date are modest. The etiology of SCOS involves a large number of causes, including Y-chromosome microdeletions, trauma, viral infections, exposure to radiation or toxins, or idiopathic causes. The seminiferous tubule may be involved in its entirety or affected in a focal pattern only, with residual islands of spermatogenesis, which explains the variability in the success rate of sperm recovery in these patients. No prognostic markers, hormonal or of other nature, are currently employed in clinical practice. The purpose of this review is to organize the known information on SCOS and define current correct diagnostic and clinical practice, focusing in the second section on areas of research to look out for in terms of potential practical developments from the vast knowledge accumulated over recent decades.

## 1 Introduction

Infertility, defined as the inability of a sexually active couple to conceive after a year of attempts, is a significant problem in our era (1, 2). According to WHO data, this issue affects at least 15% of couples living in industrialized countries (2, 3). Furthermore, about half of infertility cases are due to male partner pathologies, either alone in 20% of cases or in combination with the female partner problems in a further 30% of cases (1). Alterations in the male partner’s semen can range in type and severity, but 10-15% of patients who consult fertility clinics are classified as azoospermic (4), meaning they have a total absence of sperm in their ejaculate. Azoospermia is further classified as obstructive (OA), in which alterations in the testicle or seminal ducts block the passage of sperm, or non-obstructive (NOA), in which the spermatogenic process is altered at various stages (5). NOA can be induced by hypospermatogenesis (HS), arrest of sperm maturation at one of the stages of the process (MA) or Sertoli cell-only syndrome (SCOS) (5). This condition was first described in 1947 (6) and represents a form of stem cell aplasia, in which the seminiferous tubules are populated only by Sertoli cells. Despite its significant frequency, affecting the majority of patients with NOA and reaching percentages of over 50% in some studies (7), this syndrome still has many unknown aspects. In recent decades, numerous conditions that can cause SCOS or SCOS-like phenotypes have been studied; therefore, it may appear that the pathology is a common outcome of numerous etiological factors rather than a separate entity. Nevertheless, the majority of patients do not have a clear etiological cause, and gene mutations or alterations in intracellular pathways have been found to be quite common in patients with SCOS, albeit with different etiologies. At the present time, research therefore appears to be focused on three main objectives: 1) finding one or more common aetiologias for cases of SCOS without a clear cause, 2) determining useful markers for the diagnosis and prognosis of the condition regarding fertility potential, 3) improving current assisted reproduction techniques for these patients and beginning to experiment with alternative methods for spermatogenesis recovery, where possible.

This narrative review aims to reorganize and simplify the information known to date on SCOS, with a significant emphasis on clinical aspects and current treatment guidelines. The second part focuses instead on the various lines of research and experimental therapies currently underway and explains the approach to researching genetic causes.

## 2 Part 1: current clinical, diagnostic and therapeutic approaches

### 2.1 Primary and secondary SCOS: histology of the seminiferous tubules

**Figure 1:**
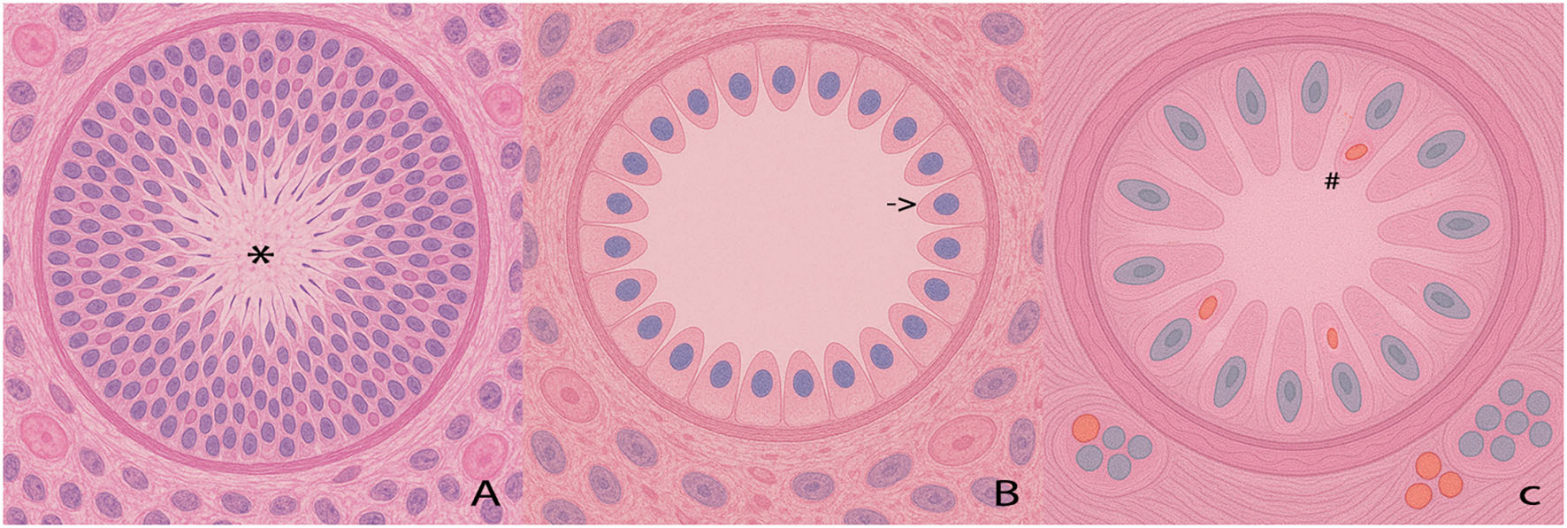
Schematic representation of seminiferous tubule sections. On the left (panel **A**) is a normal tubule. Marked with * are germ cells in different stages of spermatogenesis, which occurs in a ’spiral‘ pattern in the human testicle. In the center (panel **B**) is a picture of type 1 SCOS. Marked with an arrow; are the classic ovoid nuclei of immature Sertoli cells; note also the normal thickness of the basement membrane. On the right (panel **C**) is a picture of type 2 SCOS, showing a thickening of the basement membrane and an increase in collagen fibers. Marked with # are the intracellular lipid vacuoles.

The first distinction that needs to be clarified regarding SCOS is the presence of two different histological entities, defined as primary and secondary, which were first identified in 1991 (8). Primary SCOS is considered a complete form (cCOS), in which germ cells are completely absent from the testicular lumen; its genesis appears to occur prenatally due to an error in gonocyte migration (8, 9). Patients with primary SCOS have no foci of testicular spermatogenesis. Histologically, the seminiferous tubules are reduced in size due to the lack of gonocytes, with walls of normal thickness, and are populated only by Sertoli cells that appear immature, showing ovoid-rounded nuclei, columnar cytoplasm and vimentin located only in the sub-nuclear area (10, 11). The secondary form of SCOS has a completely different histological appearance (See **Figure 1**), with the only common feature being the reduced thickness of the tubules. Sertoli cells in place have a post-pubertal appearance, with irregular nuclei and diffuse vimentin. The tubular walls appear significantly thickened, with the presence of peri-tubular fibrosis and thickening of the collagen fiber layer. There are also sporadic lipid granules within the cells, indicating the reabsorption of degenerated Sertoli cells (10, 11). The main difference is the presence of foci of spermatogenesis in some tubules, which allows secondary SCOS to be classified as an incomplete form (iSCOS) but which can nevertheless progress over the years to a complete form when the islands of spermatogenesis begin to diminish, perhaps also due to further damage to gonadal function.

The existence of different histological variants of SCOS, and therefore probably different etiologies, has been known since a study in the early 1990s (12). Three possible combinations of Sertoli cell types were found in the testicles of patients with SCOS, unlike biopsies in patients with other testicular damage (hypogonadotropic hypogonadism, cryptorchidism, estrogen or chemotherapy treatment), which showed a more homogeneous picture. Some patients with SCOS had only normal mature Sertoli cells, which is consistent with a secondary form, others had a mix of different types of pre-pubertal or intermediate disgenetic Sertoli cells, and some had a mix of mature and involuted Sertoli cells, with clear signs of cell damage. The latter were predominant in patients treated with chemotherapy or exposed to other gonadotoxic therapies. This variability, already assessed 35 years ago, shows how complex the picture of this pathology is and how defining a pattern common to all patients is a truly complex process.

### 2.2 Approach to patients with SCOS: physical examination, hormone testing and diagnostic procedure

Patients diagnosed with SCOS are typically males between the ages of 20 and 40 who seek medical attention for infertility (13). The clinical history, phenotype and physical examination may vary depending on the etiology of SCOS and in some cases be very characteristic (e.g. forms linked to Klinefelter syndrome (14)), but in most cases where the etiology is unclear, these patients show normal secondary sexual characteristics, signs of normal virilization and absence of gynecomastia. On palpation, the testicles are generally in place, of normal shape and consistency but reduced in volume to the point of severe hypotrophy (13).

Testicular ultrasound does not allow a definitive diagnosis of SCOS. However, some characteristics such as reduced testicular volume and reduced volume of the epididymal head are common in patients with NOA (15).

Obtaining a correct medical history that includes exposure to toxic substances, previous radio- and chemotherapy, cryptorchidism corrected in childhood, previous severe varicocele treated surgically, or testicular trauma is essential (16), as these conditions can all cause secondary SCOS (17–25). To classify patients as azoospermic, two semen analyses must be performed at least 90 days apart, as indicated by the guidelines (1, 3, 26, 27), to allow time for a full new cycle of spermatogenesis to be completed.

**Figure 2:**
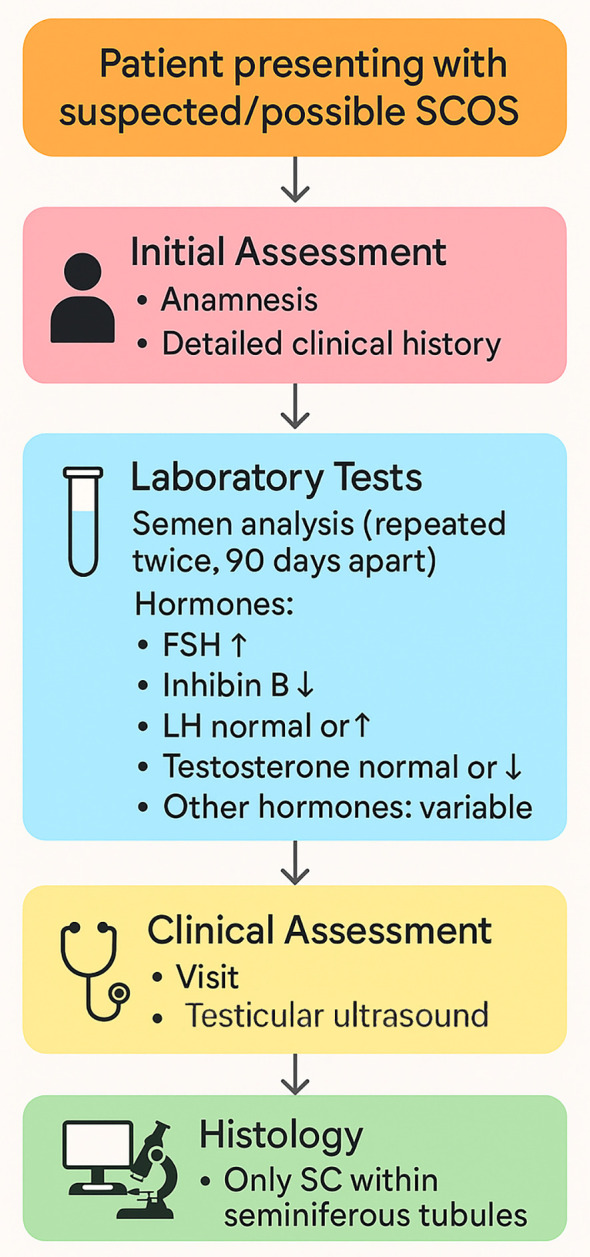
Simplified flowchart showing the initial approach to a patient with suspected/possible SCOS. A thorough medical history, physical examination and testicular ultrasound are essential during an initial andrological consultation.

Hormone testing is routinely performed in azoospermic patients (16), and elevated FSH levels are almost always associated with alterations in spermatogenesis (28). In most patients with SCOS, FSH levels are elevated (29, 30), while the expression of its receptors and its bioactivity appear to be within normal limits (31). The increase in FSH seems to be due to a dysfunction of Sertoli cells, mainly in immature or involuted forms, but it is also related to the reduction in levels of Inhibin B (32, 33), which is involved in the regulatory feedback of pituitary FSH secretion. Inhibin B is an important marker of Sertoli cell function and is reduced both due to lack of production and decreased entry into the bloodstream, linked to the known thickening of the tubule lamina and extracellular matrix. **Figure 2** illustrates the optimal flow chart for an initial diagnosis of SCOS.

In patients with SCOS, LH, estradiol and testosterone levels are generally normal (13), although multiple cases with complex and convoluted hormonal alterations, sometimes associated with multiple concomitant testicular pathologies, have been reported in the literature (34). In cases where LH is significantly increased, testosterone is reduced or the T/LH ratio is lower than normal, there is generally a concomitant dysfunction of the Leydig cells (35, 36), which is not uncommon in patients with NOA and often makes it difficult to understand which cell line was the first to experience damages. In the seminiferous tubules of patients with SCOS, Leydig cells are not generally increased in number, but may cluster together or become hypertrophic (12). Leydig cells in adult males are the main promoters of the conversion of testosterone and androstenedione into estradiol and estrone (20), and in cases of their dysfunction, the activity of aromatase (CYP19A1) may increase, altering the ratio between androgens and estrogens in the tubule (37, 38). This can further damage the balance of spermatogenesis. Finally, the expression of hormonal receptors on Sertoli cells in SCOS was evaluated, in particular the androgen receptor (AR) and its co-regulators (39). AR is mainly expressed by somatic cells and is increased in SCOS, probably due to the lack of germ cells. Some co-regulators are expressed in an increased (such as ARA55) or reduced (such as ARA54) fashion or even in different locations (39). The gene encoding AR has in fact been extensively studied for SCOS-related polymorphisms (40–43).

### 2.3 Testicular biopsy in patients with SCOS and promising seminal markers

The definitive diagnosis of SCOS requires a testicular biopsy showing tubules lined only by Sertoli cells without germ cells (13). The presence of two types of SCOS, primary and secondary, explains the different histological picture in biopsies. Furthermore, in secondary SCOS, focal areas of spermatogenesis may be found (44). In some cases, SCOS has been found in only one of the two testicles or in a mixed form in both gonads, which is why several authors recommend bilateral biopsies (45).

Although biopsies are now safe and routinely performed, they remain an invasive procedure, especially when used for diagnostic purposes only and not for sperm extraction (46). For this reason, for many years researchers have sought alternative semen markers that can point towards a diagnosis of SCOS or at least a form of NOA (29, 47). For example, reduced levels of laminins in seminal fluid have been proposed as a screening test for SCOS (48–50): these glycoproteins are significantly reduced in men with seminal fluid abnormalities, most notably in NOA and SCOS, reflecting impaired spermatogenesis or impaired production by Sertoli cells. Nevertheless, this marker alone does not seem enough for a diagnosis, and the concomitant use of other tests has been proposed.

For example, a 2016 study (51) evaluated the dosage of certain free seminal mRNAs to assess the expression of testicular genes. DDX4 (DEAD box polypeptide 4) can be found in both seminal fluid and testicular biopsies, and its absence appears to be related to SCOS and not to other forms of NOA. It also allows the severity of the syndrome to be quantified, distinguishing in part between complete and incomplete forms (52). The possibility of employing proteomics studies has greatly expanded this field of research, allowing the discovery of numerous other markers, including TEX101 (testis-expressed 101) and ECM1 (47), one produced by the testis and the other by the epididymis, which when combined allow for discrimination with good sensitivity and specificity between OA and NOA and, even better, between SCOS and other forms of NOA. Numerous other proteins appear promising for the diagnosis and prognosis of various forms of azoospermia, and combining them makes them even more useful (29, 47). However, it should be emphasized that their use is currently only experimental.

### 2.4 Genetic analysis and patient counselling

The genetics of SCOS are extremely complex (14, 53–55). In recent decades, the involvement of dozens of genes has been hypothesized, due to their amplifications, deletions or single nucleotide polymorphisms. In addition, epigenetic processes also appear to be related. Advances in technology have enabled genomic studies to simultaneously evaluate multiple genes and the presence of copy number variation in patients with NOA. Nevertheless, to date, most of these etiological hypotheses, although well-founded, are not part of the normal clinical tests for patients. However, there are some well-defined and very common genetic causes in patients with SCOS, which should be routinely excluded in every recently diagnosed patient (13, 14).

Karyotype abnormalities are quite common in these patients. The most common is Klinefelter syndrome (14, 53), which affects 1 in 500 males at birth (56). The severity of azoospermia in patients with Klinefelter syndrome varies, as mosaicism is seen in many cases. These patients show obvious signs and symptoms on physical examination, especially in forms without chromosomal mosaicism, including marked testicular hypotrophy, gynecomastia and eunoicoid appearance (56). The probability of obtaining sperm from the testicles of patients with SCOS linked to the 47/XXY karyotype is not so different from other causes, as we will see below (57).

Another karyotype associated with SCOS is 45 X/46 XY mosaicism (58), of which sporadic clinical cases have been reported in the literature. The phenotype and testicular impairment, up to a clear picture of SCOS, depend, as always, on the number of cells showing the alteration. Rare cases of SCOS have also been reported in patients with karyotype 46/XX (59), in whom, due to unequal crossing over during paternal gametogenesis, the SRY gene was transferred to the short arm of the X chromosome and thus inherited by the offspring with female karyotype 46/XX.

In addition to alterations in chromosome number, translocations and inversions are also known to cause problems with male fertility (55). For example, males presenting with a reciprocal translocation of the X or Y chromosomes may experience spermatogenic arrest or other testicular issues. The most common Robertsonian translocation associated with male infertility is t(13q14q) (55). Another genetic cause that is routinely investigated in azoospermic patients is the alteration of the AZF (azoospermia factor) locus present in the long arm of the Y chromosome (Yq11) (53, 55, 60, 61). Microdeletions of this locus have been known since the 1970s to be a source of male infertility, with a phenotype that can range from oligospermia to azoospermia with SCOS. The wide clinical variability depends both on the extent of the deletions and their location, as AZF is sorted into several regions, originally called AZFa, AZFb and AZFc (55). In the following decades, improvements in the mapping of Yq11 allowed for further subdivisions, localizing the alterations in five different regions containing different genes (including Deleted in Azoospermia- DAZ (60–64) and RNA Binding Motif, Y-linked- RBMY (55, 63, 65, 66), among the first to be studied) and having variable clinical impacts. Overall, AZF deletions appear to be found in approximately 8-15% of patients with NOA (67), while the percentage is lower in patients with severe oligospermia and is almost zero in patients with a sperm count above 5 million/ml (27). Deletions in the AZFa region are the most severe and almost always lead to SCOS, although some patients with partial deletions involving only one gene (like Ubiquitin-Specific Peptidase 9 -USP9Y or DEAD-Box Helicase 3, Y-linked - DBY) may present with cryptozoospermia (14, 55, 60). Deletions in AZFb often lead to azoospermia or cryptozoospermia, while in some cases they cause an arrest of spermatogenesis maturation (65, 66). Again, partial forms are less severe, showing usually a severe oligozoospermia. Deletions in the AZFc region, which houses the Deleted in Azoospermia genes, proximal DAZ1/2 and distal DAZ 3/4, and Chromodomain-Y linked CDY genes (61, 62, 68, 69), are historically considered less severe, especially if they are small and partial. Nevertheless, there are studies indicating that even these microdeletions can worsen the outcomes of assisted reproduction techniques. Extensive forms of deletions involving multiple regions are, as can be expected, more serious (63).

Performing a karyotype assessment and testing for the most common AZF deletions is crucial both to provide a prognosis regarding the de facto probability of sperm recovery and assisted reproduction procedures success and for genetic counselling purposes (13, 16, 28, 54). Some alterations, especially extensive deletions of certain AZF regions, render the probability of procreation almost nil, while other microdeletions, especially AZFc, may allow procreation and may therefore be transmitted to offspring. For this reason, it is essential to inform the patient and schedule a genetic consultation before proceeding with following approaches.

### 2.5 Therapeutic approaches

To date, there is no cure for SCOS that allows normal spermatogenesis to be restored. However, there are several therapeutic approaches aimed to improve the patient’s well-being and, above all, to try to achieve fertility. First and foremost, it is advisable to try to treat the possible reversible causes of a type 2 SCOS phenotype, for example, surgical correction of a severe varicocele (22, 23). The impact of this procedure on the likelihood of subsequent sperm retrieval is variable and, although there are reports of favorable outcomes, it is known that the SCOS phenotype benefits least from treatment compared to patients with varicocele and hypospermatogenesis or with varicocele and late arrest of spermatogenesis (LMA) (70). The degree of varicocele also has a significant impact on the expected outcome (22, 70).

In cases where the patient wishes to procreate, the best approach is almost always micro-TESE, i.e. sperm extraction from the testicle performed by an experienced surgeon using an intraoperative microscope (46, 71–73). In the past, normal TESE, which requires a larger amount of testicular tissue, or TESA, in which sperm were aspirated with a needle, were also routinely used (72, 74–78). Current approaches include mapping of the areas of testicular spermatogenesis and bilateral distribution of extractions to avoid excluding portions of the testicle possibly containing sperm (79).

Improvements in surgical techniques have led to an increase in the success rates of sperm retrieval, but sadly this is not applicable to the entire patient cohort. The success rate varies according to both etiology and testicular histology, being lower for cases of idiopathic SCOS (57, 80). Patients with extensive AZFa or AZFb+c deletions have virtually no chance of success (13, 62, 63), and this must be explained to the patient during counselling before proceeding with TESE. The average success rate of sperm retrieval varies across studies, but fluctuates around 37%, considering all forms of SCOS (57).

However, the search for pre-operative markers to estimate the probability of success before performing sperm retrieval is still ongoing (69). The absence of the aforementioned seminal marker DDX4 is linked to complete SCOS and therefore predicts a negative outcome of sperm retrieval (51).

Advanced imaging studies, which may include both specialist-performed ultrasounds to assess the appearance of the various tubules (79, 81) and more complex techniques (82), may be useful in predicting the success of TESE. For example, MRI with diffusion sequences to study apparent diffusion coefficient (ADC) and magnetization transfer ratio (MTR) appear to be promising tools, as ADC is significantly increased in testicles with lower cellularity and a lower probability of sperm recovery (83). Other parameters have been found to be more controversial, primarily FSH value, patient age, and testicular volume (9, 84–87). Although in some case series these parameters have been linked to the potential success of TESE, reviews of the literature show conflicting data (69, 88), concluding that actual testicular histology and etiology of NOA are ultimately the single most important factors. Once sperm has been obtained, the success of ICSI in patients with SCOS appears to be comparable to that of other patients with NOA, with approximately 13% of patients becoming biological fathers in the end (57, 80, 89).

**Figure 3:**
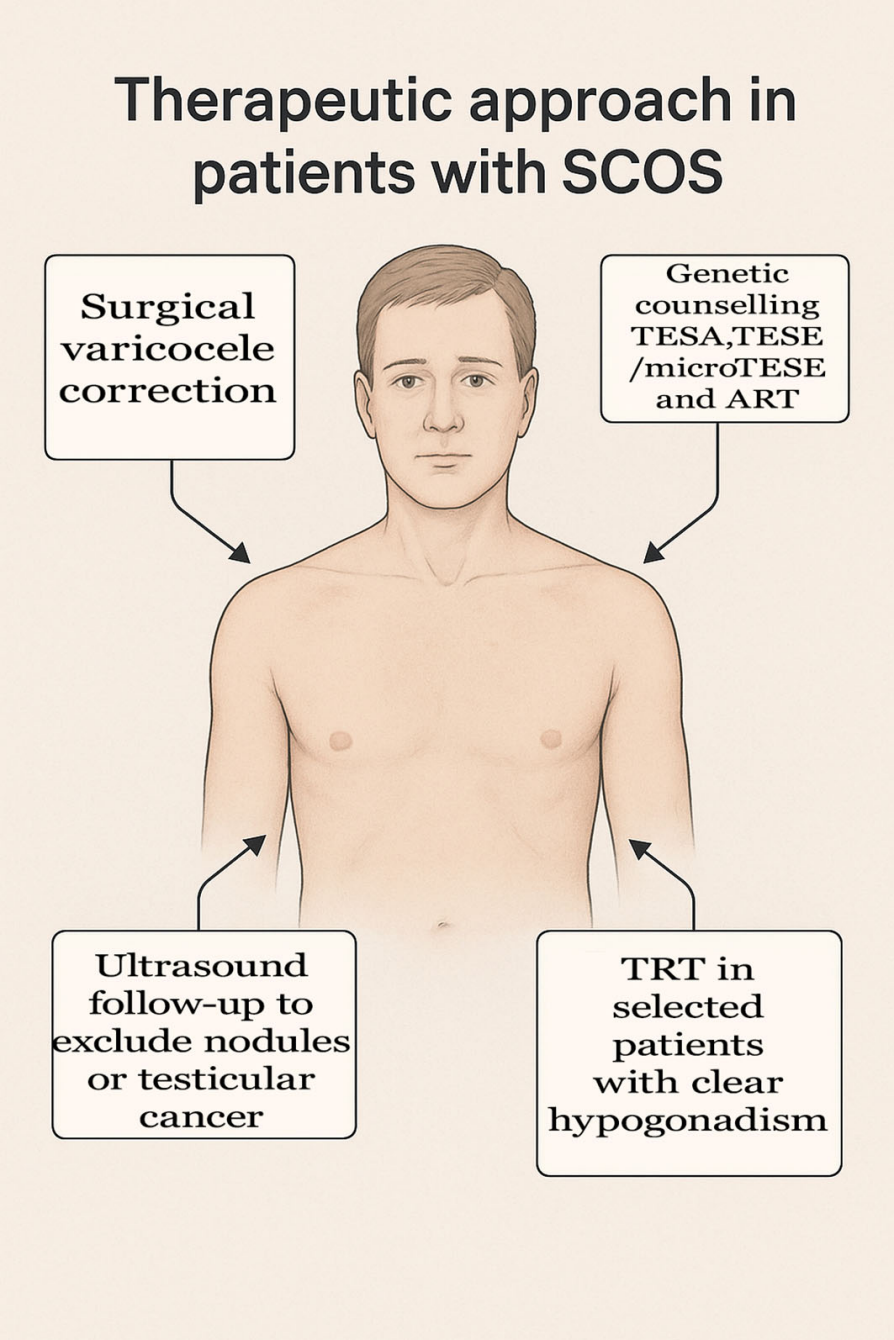
Summary of therapeutic approaches. Attempting assisted reproduction is not the only clinical need for SCOS patients, and this must always be taken into account by physicians who treat these patients.

Clinicians treating patients with SCOS must also always remember that they show an increased risk of developing testicular neoplasms (90–92), both malignant (germ cell tumors, lymphomas) and benign (nodules, Leydig cell hyperplasia), so scheduling an adequate ultrasound follow-up is essential. In patients with concomitant Leydig cell dysfunction and hypergonadotropic hypogonadism, testosterone replacement therapy should be considered based on the clinical situation, age and desire to have children (93). **Figure 3** summarizes current therapeutic approaches.

## 3 Part 2: future diagnostic and therapeutic approaches

### 3.1 Research into the genetic causes of SCOS

In addition to Klinefelter syndrome and AZF deletions, many other genetic causes have been evaluated to explain the etiology of SCOS in patients where it is unclear (14, 53, 54, 94).

This field of study appeared difficult right from the start, for multiple reasons. First, there are thousands of genes involved in spermatogenesis (95), and this process is extremely complex. For years, the main approach has been to search for mutations, deletions or single nucleotide polymorphisms (SNPs) in genes known to be associated with infertility in patients with SCOS, comparing the results with fertile patients. Considering that at least 1450 alleles linked to male infertility in mice are known to date (96–98), the road has always been difficult. Furthermore, mutations that induce infertility are not widespread in the population, for the obvious reason that they are not transmitted to the next generation. However, there are alterations that cause incomplete forms of SCOS and are sometimes inherited or grouped in small family clusters. In such cases, whole-exome sequencing (WES) (99) combined with mapping of homozygous regions and linkage studies have yielded interesting results. With the advancement of methods and the introduction of massive parallel sequencing and the expansion of online databases to identify coding portions, such as ORFs, much more powerful tools have begun to be used to identify SNPs or copy number variations (CNVs) (14, 52, 100, 101) by comparing large fragments of the genome of SCOS patients with fertile controls. Going further and defining which variations are linked to the phenotype and which are sporadic and meaningless has not been easy, as many CNVs were related to genes not known for their role in infertility or not expressed in the testicles at all. In other studies, however, CNVs relevant to the SCOS phenotype were actually found on the X chromosome, mainly linked to the DUP1 gene (101), which plays an important role during spermatogenesis, but also on other chromosomes, including 12, 3 and 8. A microarray analysis of 37 SCOS patients found CNVs in other genes potentially involved in human spermatogenesis, but with no counterpart in the animal model and therefore significance not yet well defined (102).

**Figure 4:**
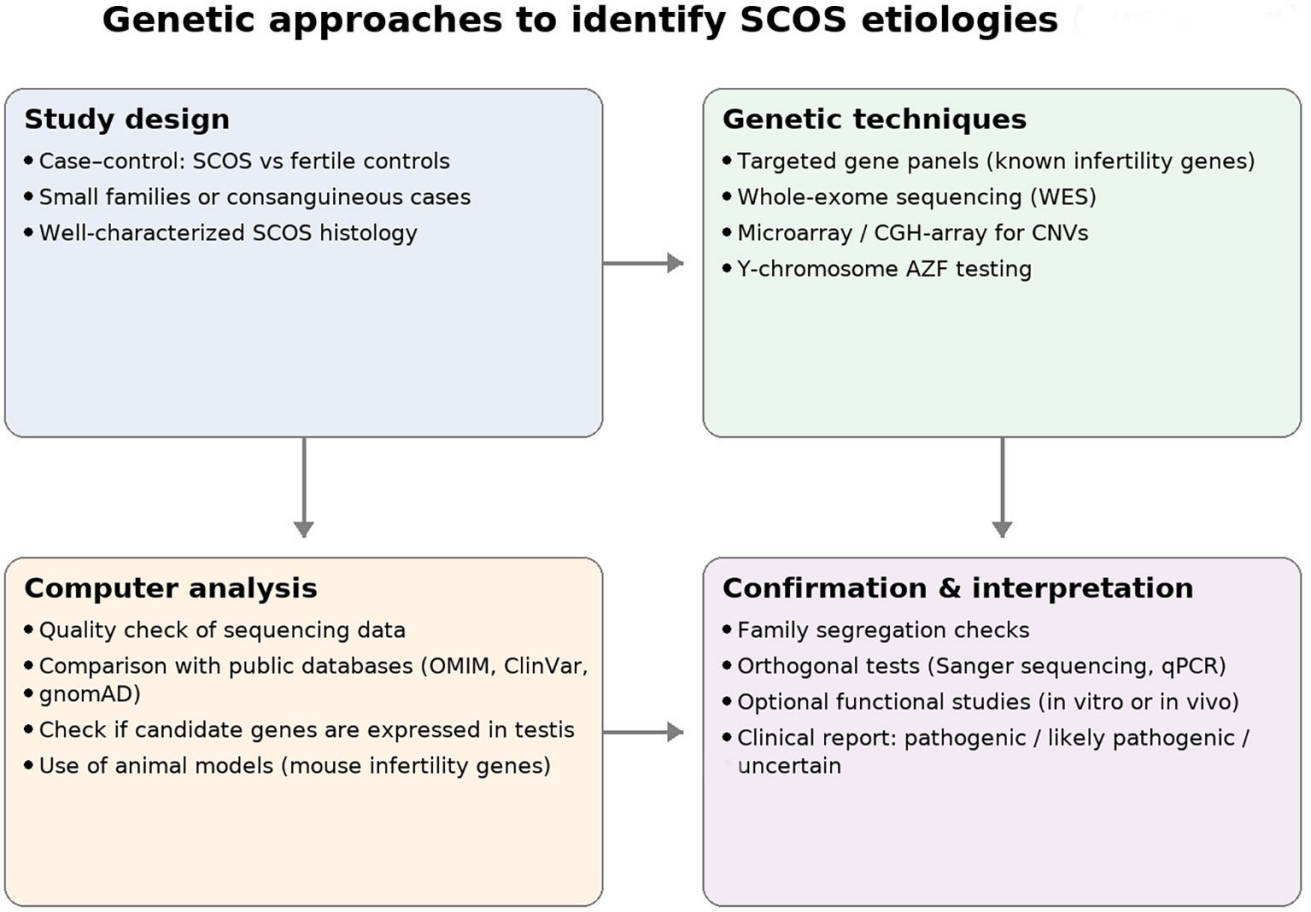
Genetic approaches to the study of Sertoli cell-only syndrome. The diagram summarizes the main stages of investigation: study design (case-control cohorts, consanguineous families), sequencing and microarray techniques, bioinformatic analysis with comparison to databases and mouse models, and finally confirmation and clinical interpretation. The integration of these steps allows the identification of genetic variants potentially responsible for SCOS.

Finding monogenic causes is thus clearly complex. WES has gradually made it possible to find genes with mutations in patients with NOA, and some are clearly associated with the spermatogenic process and also expressed in the mouse model. However, the vast majority are not exclusive to SCOS and are also present in other forms of NOA with some overlap. Many genes have been hypothesized to be involved in the pathology (103), but some seem more promising candidates than others. **Figure 4** illustrates the genetic approach that can be applied to identify new genetic alterations associated with SCOS.

A 2018 review (104) identified 78 genes related to male infertility, but only two were convincingly linked to the SCOS phenotype, both of which are autosomal recessive, namely FANCA and FANCM.

These two genes, linked to the development of Fanconi anemia, are respectively located on chromosomes 16 and 14, and there is considerable scientific evidence relating them to SCOS, as they are involved in spermatogenesis, their variants have been shown to be clearly pathological, and they are also being studied in animal models (105). A homozygous variant of FANCA was found in two brothers with SCOS and Fanconi anemia (106), and subsequently a further pathogenic heterozygous variant in another unrelated patient. Screening for this mutation in an azoospermic population could help diagnose Fanconi anemia in adult patients with fewer signs and symptoms of this disease. In later studies, further variants of the gene have been identified in other patients with NOA (107).

Over the years, even more data has been accumulated on FANCM (105, 108, 109): it is an essential gene for ensuring the stability of genetic material during spermatogenesis, especially in the early stages, and is well studied in the mouse model. The first study on this subject was conducted in 2018 (105) and found loss-of-function (LOF) mutations in heterozygosity in two Estonian brothers with SCOS, and subsequently several nonsense mutations in homozygosity in two other unrelated patients. In the testicles of these patients, testicular expression of FANCM was minimal or completely absent. During following years, other different heterozygous or homozygous mutations were detected in patients with SCOS phenotype, and in 2024 also in a patient with concomitant SCOS and diffuse astrocytoma (110).

In women, FANCM and FANCL mutations have been associated with premature ovarian failure (111).

AR related three-nucleotide polymorphisms, such as GGN and CAG, have also been investigated extensively (40–43). The data appeared controversial, but a 2016 meta-analysis (42) supported the hypothesis that increased CAG repeat length is linked to infertility and azoospermia. Proper AR function is obviously essential for spermatogenesis, since the hormonal level inside the male gonad is crucial.

GILZ (X-linked gene glucocorticoid-induced leucine zipper) is another interesting gene related to the SCOS phenotype, extensively studied in rats (112–115) but also expressed in humans, which is responsible for regulating the well-being of spermatogonial stem cells (SSCs) and the inflammatory state of the testis. In one study, numerous GILZ polymorphisms were noted in 65 patients with SCOS in Australia (116), discovering new variants, but these are unlikely to have a high prevalence in the general population.

Variants of MCM9, a gene essential for DNA repair, had already been associated with hypogonadism in men (117). Its alteration is also a known cause of spermatogenesis alterations in mice, and a recent study (118) identified two new homozygous LOF mutations in two patients with SCOS, in whose testicles MCM9 was completely absent. Given the wealth of data on its importance in spermatogenesis, this gene also appears to be a promising candidate.

HELQ is another gene involved, essential for the repair of double-stranded DNA breaks, and its alteration is also known to cause infertility in mice (119). WES of 20 patients with SCOS phenotype determined the existence of two new missense mutations in heterozygosity (M1 and M2) in two Chinese patients and later four other different mutations (M3-M6) in another cohort of patients. Its impact on the general population has yet to be determined (120, 121).

Mutations in the NANOS2 gene are known on animal models for their negative effect on spermatogenesis (122–124). This protein regulates many metabolic pathways required for the survival of SSCs. It is expressed only in male primordial germ cells (PGCs) during development and later in SSCs.

Through WES in eight consanguineous families in the Middle East with NOA, pathogenic recessive variants of several genes, including NANOS2, were found (99). Two brothers with SCOS showed the missense variation p.Gly70Arg, while another subject had an additional mutation, but linked to a phenotype of arrested spermatogenesis maturation (99).

**Figure 5:**
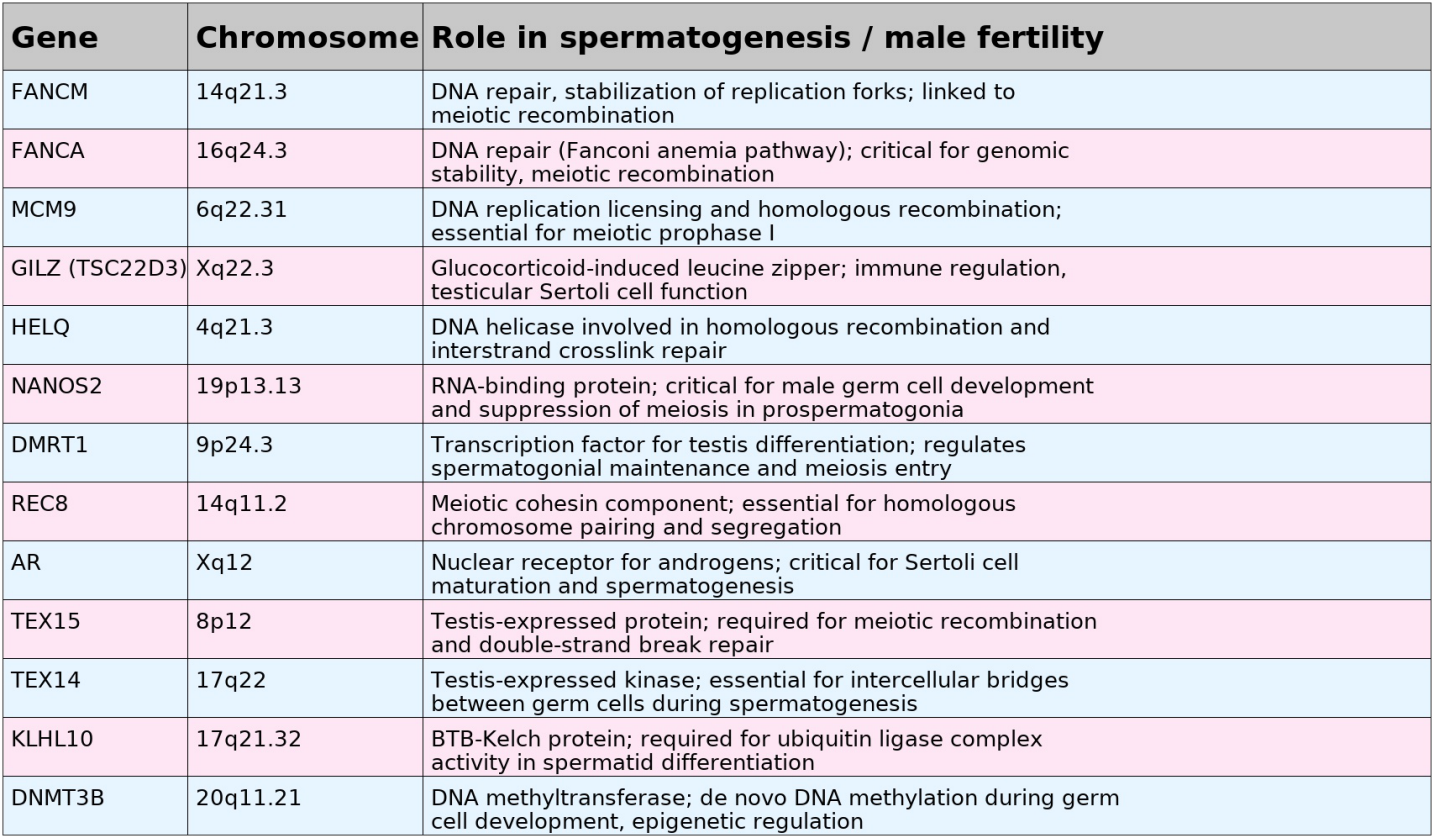
List of the main genes investigated in patients with SCOS phenotype, their chromosomal location in humans and the action of the encoded protein.

At least seven other potential genes related to the SCOS phenotype were identified or confirmed in a 2019 study (125). Among the most promising: two variants in Doublesex and Mab-3 Related Transcription Factor 1 (DMRT1), which is known for the SCOS phenotype in mice and had already been reported in infertile men, a new variant in REC8 Meiotic Recombination Protein (REC8), which is essential for meiosis and already associated with infertility in men, as well as new variants in genes with moderate or limited evidence of correlation with the SCOS phenotype, but with existing data on animal models or other types of NOA (TEX15, KLHL10, DNMT3B and TEX14, See **Figure 5**).

### 3.2 Research on gene expression and alteration of signaling pathways in patients with SCOS

New methods for studying gene transcription and protein production, such as microproteomics (126), as well as the study of epigenetics, have provided a better understanding of which mechanisms are actually altered in SCOS. These tests are generally performed on testicular tissue from diagnosed patients, therefore without stem cells: as a result, they mainly provide information on the gene expression of Sertoli cells. Sertoli cells are essential for proper spermatogenesis, as they perform physical support, immunological protection and growth factor production functions (127). The collaboration between the various testicular cells is very complex, and some studies have attempted to investigate the role of each individual cell by evaluating the total transcriptome of somatic cells (128). The study of the secretions produced by Sertoli cells has provided insight into their actual usefulness, showing their crucial role in both proliferation and the control of pro- and anti-inflammatory stimuli (129). By comparing the entire transcriptome of Sertoli cells from healthy patients with those from SCOS, it has been possible to evaluate the main altered pathways, which primarily include the formation of the blood-testis barrier (BBB) and proliferation, involving production of GDNF, FGF8 and BMP4 (130).

The BTB is essential for enabling communication between cells and interaction with SSCs, while protecting the spermatogenic niche from toxic or harmful factors and allowing the selective passage of only certain small molecules, such as secondary messengers (131–133). The expression of certain proteins involved in BTB formation, including Claudin 11 (134), JAM3, Nectin 2 and certain Cadherins, was the same in the tissue of patients with SCOS or even increased when compared with fertile patients, while GJB2, which codes for Connexin 26, was markedly reduced (130). At the same time, the expression of some spectrins (SPTB, SPTBN4, and SPTBN5) appeared to be significantly reduced: the lack of these proteins compromises the correct distribution of junction proteins and the polarity of the cytoplasm, creating a ‘disorganized’ Sertoli cell that is unable to direct proteins in the correct direction. A recent microproteomics study (126) confirmed this hypothesis, demonstrating that the SPARC protein is under-expressed in the Sertoli cells of patients with SCOS, leading to reduced expression of multiple genes related to cell adhesion (FN1, ITGA5, COL6A1, VWF, LAMB1 and THBS1) and some proteins essential for BTB formation and signal transmission through gap junctions, namely GJA1, CDH1, and ZO-1.

There is extensive literature on the altered expression of growth and cell proliferation factors in SCOS patients, especially with regard to Glial cell line-derived neurotrophic factor (GDNF). GDNF mRNA expression is reduced in SCOS up to 20% of normal values. The testicle with SCOS phenotype also expresses much less GFRA1, the ligand-binding domain of the GDNF receptor (135). These values are not sufficient to ensure stem cell development. GDNF acts synergistically with HGH (Hepatocyte growth factor) and its role is also fundamental in *in vitro* cultures (136). The mRNA expression of some members of the FGF family has also been found to be reduced in SCOS, in particular FGF8 (130) and FGF5 (137), compared to patients with OA. Both factors are essential for maintaining the correct integrity of the spermatogenic niche (138) via ERK and AKT activation. There is concrete evidence that other pathways may also be altered, such as TGF-beta and Wnt/β-catenin signaling (139).

Another pathway that is clearly altered in the SCOS phenotype is apoptosis. Proper management of programmed cell death is necessary for spermatogenesis to occur. In SCOS patients, several pro-apoptotic factors are altered. Fas, Fas Ligand and activated caspase-3 are overexpressed in the SCOS phenotype, both in Sertoli cells and in interstitial hyperplastic cells (140), and this appears to be the main activator of apoptosis in the testis (141), especially in SCOS (142). Src-associated substrate in mitosis of 68 kD (Sam68) is also reduced in SCOS compared to OA, leading to the activation of apoptosis (143).

PL Heterogeneous nuclear ribonucleoprotein L (HnRNPL) is another example of an anti-apoptotic factor that is under-expressed in the SCOS phenotype, and its deactivation in the mouse model leads to apoptosis of spermatogenic cells (144). Recently, increased pyroptosis, mediated by increased expression of CASP1 and CASP4, has also been proposed (145) as an alternative mechanism of SCOS genesis. In the samples from this study, interleukins such IL-1 β and IL-18, LDH, and ROS, all clear markers of inflammation, were also significantly increased.

In fact, inflammation is completely uncontrolled in the SCOS phenotype. This is not surprising given the importance of functioning Sertoli cells in protecting the spermatogenic niche from these insults (127), both physically and chemically. The expression of pro- inflammatory markers, such as IL-6, TNF-α, CXCL2, NLRP3, and CCL2, is increased in NOA, and an inflammatory cell infiltrate, including macrophages (146) and mast cells (147), is often noted. The aforementioned hypothesis of hypo-expression of the GILZ gene strongly supports the possibility of an inflammatory component in the genesis of SCOS (114).

Altered gene expression in patients with SCOS is also evident when observing variations in microRNAs, which do not directly encode but are essential for regulating transcription and translation of other RNAs and whose alteration is known to be related to infertility (148), as they are essential for many of the pathways above mentioned, primarily apoptosis and cell proliferation (149). One study found 174 miRNAs differently expressed between patients with SCOS and patients with OA (150). Although interesting, the studies are currently preliminary and often performed on small cohorts (151), but miRNAs could nevertheless be a future therapeutic target (152).

### 3.3 Future therapeutic approaches

Notwithstanding the robust amount of data available and the many open lines of research, there is currently no therapy for SCOS since it’s impossible to reconstitute the population of SSCs missing in the testis. The last few decades have seen rapid progress in technologies for obtaining pluripotent stem cells (iPS) even from somatic cells, as shown by the well-known 2007 work that obtained them from human fibroblasts. Sertoli cells can also be obtained from fibroblasts (153). Subsequent studies have shown the potential to obtain SSCs from iPS (154), which then appeared capable of progressing through meiosis and generating haploid cells. These cells can then be propagated in suitable culture systems (155), but the yield in terms of number and functionality is generally insufficient for clinical application, partly because it is very difficult to recreate the complex spermatogenic niche and its support system (156, 157). Current approaches include the use of 3D scaffolds, co-culture systems, enriched cultures or the creation of organoids to best support spermatogonial cells (158–161).

A very interesting line of research is that related to Very small embryonic-like stem cells (VSELs), whose characterization has remained shrouded in mystery for at least a decade after their first identification in bone marrow (162). These small cells are pluripotent, scarce and normally remain quiescent, but they are indeed present in both human and murine testicles (163) and, above all, have been found in testicular fragments from biopsies of SCOS patients. Furthermore, they appear to be resistant to gonadotoxic treatments, as proven by their presence in the testicles of adult patients who survived cancer in childhood (164). VSELs are very promising for a possible therapeutic approach.

The spermatogonial stem cells obtained should be then transplanted into the seminiferous tubules of patients. There are many papers on this subject in animal models (165), while clinical trials are currently underway in humans, including one at the University of Pittsburgh (166), which offers a transplant of both stem cells and testicular tissue grafts. There is already promising data on autologous transplants of cells taken from bone marrow in patients with NOA (BM-MSCs) (167, 168). At present, some new practical research on ultrasound-guided techniques for inserting stem cells into the seminiferous tubules is still being published and is in the pre-print phase.

The therapeutic attempts described so far mainly apply to patients with SCOS without a clear genetic etiology. In cases where a gene mutation is present, the transplanted cells may retain the same defect therefore not restoring fertility.

In mice, the CRISPR-Cas9 technique has been shown to be useful for repairing gene mutations in spermatogonial stem cells, thus restoring fertility (169). This technique can also be employed to deactivate gene transcription at various stages of spermatogenesis, selectively targeting Leydig or Sertoli cells, allowing the mechanisms involved to be studied with precision (170). To date, no human trials related to SCOS or NOA in general have yet begun.

Finally, some studies are underway regarding the possibility of intervening on the spermatogenic niche rather than on the spermatogonial cells themselves, with both preclinical and clinical approaches, such as the use of intra-testicular injections of platelet-rich plasma in patients with NOA, especially after failed microTESE (171, 172). Several carriers are being studied to deliver growth factors or proteins useful for spermatogenesis to the spermatogenic niche, such as lipid nanoparticles, small extracellular vesicles (sEVs) or exosomes (19, 173, 174). These molecules share low immunogenicity and the ability to move easily through biological barriers, even reaching the protected site where spermatogenesis occurs.

## 4 Conclusion

Sertoli cell-only syndrome is a relatively common condition in infertile men, but it has dozens of known causes and many more that are still to be clarified. The overall result of genetic and epigenetic alterations or the impact of interfering environmental factors leads to a common phenotype of total (in the complete form) or almost total (in the partial form) absence of spermatogonial cells, rendering the probability of procreation very low. Improvements in sperm retrieval techniques have certainly helped many patients become biological fathers, but as far as new diagnostic, prognostic and therapeutic approaches are concerned, the great strides made in research have not yet been translated into clinical practice. SCOS is perhaps one of the conditions in which the gap between gathered knowledge and improvement in prognosis is widest. Clinicians need to be aware of the current evidence-based approach for the best treatment of patients with this condition, but at the same time, we need to wait to be able to take full advantage of the new tools that will be available in the near future.
